# Optimizing Ammonia Detection with a Polyaniline−Magnesia Nano Composite

**DOI:** 10.3390/polym16202892

**Published:** 2024-10-14

**Authors:** Sharanabasava V. Ganachari, Fatheali A. Shilar, Veerabhadragouda B. Patil, T. M. Yunus Khan, C. Ahamed Saleel, Mohammed Azam Ali

**Affiliations:** 1 Center for Energy and Environment, School of Advanced Sciences, KLE Technological University BVB Campus Vidyanagar, Hubballi 80031, Karnataka, India; 2 Department of Civil Engineering, Jain College of Engineering, Belagavi 590014, Karnataka, India; shilarone@gmail.com; 3 Institute of Energetic Materials, Faculty of Chemical Technology, University of Pardubice, 53210 Pardubice, Czech Republic; iamveerabhadraa@gmail.com; 4 Central Labs, King Khalid University, AlQura’a, Abha P.O. Box 960, Saudi Arabia; yunus.tatgar@gmail.com (T.M.Y.K.); ahamedsaleel@gmail.com (C.A.S.); mazam@kku.edu.sa (M.A.A.); 5 Department of Mechanical Engineering, College of Engineering, King Khalid University, Abha 61421, Saudi Arabia

**Keywords:** nanocomposites, polyaniline magnesia, in-situ oxidative polymerization, Fourier transform infrared spectroscopy, ammonia sensing

## Abstract

Polyaniline−magnesia (PANI/MgO) composites with a fibrous nanostructure were synthesized via in situ oxidative polymerization, enabling uniform MgO integration into the polyaniline matrix. These composites were characterized using FTIR spectroscopy to analyze intermolecular bonding, XRD to assess crystallographic structure and phase purity, and SEM to examine surface morphology and topological features. The resulting PANI/MgO nanofibers were utilized to develop ammonia (NH_3_) gas-sensing probes with evaluations conducted at room temperature. The study addresses the critical challenge of achieving high sensitivity and selectivity in ammonia detection at low concentrations, which is a problem that persists in many existing sensor technologies. The nanofibers demonstrated high selectivity and optimal sensitivity for ammonia detection, which was attributed to the synergistic effects between the polyaniline and MgO that enhance gas adsorption. Furthermore, the study revealed that the MgO content critically influences both the morphology and the sensing performance, with higher MgO concentrations improving sensor response. This work underscores the potential of PANI/MgO composites as efficient and selective ammonia sensors, highlighting the importance of MgO content in optimizing material properties for gas-sensing applications.

^‡^ Current affiliation: Division of Research and Development, Lovely Professional University, Phagwara 144411, Punjab, India.

## 1. Introduction

Since the discovery of conductivity in conducting polymers by Alan Mac Diarmid and his colleagues in the 1960s, nanocomposites have experienced a significant increase in popularity. It was the discovery of a new area of polymers in science and technology, known as conducting polymers, that sparked this breakthrough in the field of polymers [1,2,3]. Dopants, including any intrinsic semiconductors, were used to increase the conductivity of the material. When it comes to polymers, the inclusion of dopants can help to remove π–π stacking and improve conductivity in a polymeric chain. While macro-structures proved to be stable, they were only marginally useful in doping and for a few other applications [2,3,4]. The discovery of nanoparticles paved the way for advancements in science and technology, as well as a greater understanding of the mass of the entire planet by scientists. In contrast to the macro-structure, which has a specific set of characteristics, the nanomaterial has a wide range of characteristics [5,6,7,8].

When a polymer is doped, the dopant produces exceptional results. Many conducting polymers have relied on gas and chemical sensing because of their flexibility with temperature range, and their ability to sense the environment has allowed them to become popular [4,9,10,11]. Conducting polymers that contain nanoparticles are more heat-conductive. Among the contributions to polymer development is a review paper by Gordon Armstrong and his colleagues on polymer nanocomposites. Their paper reviewed several attributes, such as chemical and crystal properties and applications in polymer-based nanocomposites. These concepts have been explored at comparable scales in [12,13,14,15].

An investigation into the chemical sensors of unstable organic mixtures and an overview of the results has been conducted. This information was used to determine our study’s most effective classification system for categorizing chemosensory data, resulting in four major categories. The technologies used in this research were spectrometry, chromatography, and electrochemical, mass, and optical sensors. Further, electrochemical and fiber-optic technologies are the most promising long-term sensors available [16,17,18,19]. The gas sensors were based on titanium dioxide nanoparticle fiber chemiresistors with PEDOT and PANI layers. The characteristics of the two mixtures were inconsistent in their physical properties when grown in warmer and more humid conditions. A downward slope in the I–V curve was observed for the PEDOT-based sensor. However, when the humidity level was raised, the I-V slope of the PANI sensor became more favorable. It has been demonstrated that the use of humidified catalyzed gas results in a reduction in the amount of time required to respond as well as an improvement in the chemical that is detected by the sensor [20,21,22,23,24,25,26,27,28,29,30,31]. PANI and its various doped forms have been studied for their ability to sense energetic materials. Some protonic acids and Fe-nanoparticles have been used. Fe-doped PANI was more selective towards traditional energetic materials like CL20, TNT, RDX, and PETN. This study shows that PANI and its doped forms can sense all energetic materials with optimum LOD and LOQ limits [20,21,22,23,24,25]. 

Recent research has found that the sol-gel technique and PANI nanofibers can create zinc oxide (ZnO) nanoparticles. Electrospinning has been used to fabricate PANI/ZnO nanocomposites [26,27,28]. Using variations in chemoreceptor resistance, it is possible to identify the adsorption of HCl and NH_3_ at STP. When exposed to ammonia vapor, the resistance of the sensors increases, but when exposed to HCl vapor, the resistance decreases [29,30,31,32,33]. PANI has shown a greater sensitivity to breakage after being used in a PANI/ZnO nanocomposite than native PANI [34,35,36]. When it comes to electrical conductivity, the PANI/AgO sensors that have been developed perform well and have a form factor of 0.8 for signals in the 100–200 MHz range. Chemically synthesized copper nanocomposites (CuNCs) have been used as a chloroform sensor for vapor concentration levels in the parts per million range [37,38,39,40]. It is claimed that this device will be able to imitate the processes of chloroform absorption and desorption on ionized metallic surfaces. The device has previously been created. A wide range of chemical sensors constructed from this metallic cluster and conducting polymers can be utilized effectively [28,41,42,43,44].

Building on the identified challenges in ammonia detection, the current work focuses on developing and characterizing a polyaniline-magnesia (PANI/MgO) composite for enhanced ammonia sensing. The primary objective of this study is to address the limitations of existing sensors by creating a material that offers improved sensitivity, selectivity, and response time, even at room temperature. The methodology employed involves the in-situ oxidative polymerization of aniline to synthesize the PANI/MgO composite, followed by a comprehensive characterization using Fourier Transform Infrared Spectroscopy (FTIR), X-ray Diffraction (XRD), and Scanning Electron Microscopy (SEM). These techniques are utilized to analyze the molecular structure of composite, crystallinity, and surface morphology, respectively. Subsequently, the PANI/MgO composite is applied to develop ammonia gas-sensing probes, with performance evaluations conducted under controlled conditions to assess the material’s efficacy. This study explores the fundamental properties of the PANI/MgO composite and demonstrates its practical application in real-world ammonia detection scenarios.

### Advantages of Our Sensor Material and the Need for Developing New Sensors

Our sensor material, based on a polyaniline−magnesia (PANI/MgO) nanocomposite, offers several key advantages over existing commercial sensors. Firstly, the synergistic interaction between polyaniline and magnesium oxide nanoparticles enhances the sensitivity and selectivity of ammonia detection, even at very low concentrations. Our sensor material demonstrates superior performance at room temperature, making it more energy-efficient than others requiring elevated temperatures to function optimally. The PANI/MgO composite also shows rapid response and recovery times, critical for real-time monitoring applications.

Despite the availability of commercial sensors, there is a continuous need for innovation in sensor technology due to the evolving demands of various industrial and environmental applications. Existing sensors often face limitations such as insufficient sensitivity at low concentrations, slow response times, and the requirement for complex and expensive fabrication processes. Moreover, many commercial sensors lack specificity, leading to false positives when detecting ammonia in the presence of other gases.

The development of our PANI/MgO composite sensor addresses these gaps by offering a more reliable, cost-effective, and easy-to-fabricate alternative. It provides a highly selective detection mechanism, minimizing interference from other gases, which is particularly beneficial in environments with complex chemical mixtures. Furthermore, our sensor material is developed using a green synthesis approach, aligning with the global push towards sustainable and environmentally friendly technologies. In summary, while many ammonia sensors are available, our research contributes a novel material that surpasses existing technologies in critical performance metrics, addressing specific limitations and meeting the growing need for advanced sensor solutions in diverse applications.

## 2. Experimental

### 2.1. Materials

Aniline solution, sulfuric acid, hydrochloric acid, ammonium persulfate crystals, and ferric chloride crystals were purchased from Sigma Aldrich, Chemicals Private Limited, Bangalore, Indian (analytical grade). The aniline was dissolved in HCl. Ammonium persulfate (APS) crystals were dissolved in water to achieve the desired concentration. All the solutions were made to 0.1 N during the experimentation. Coming to MgO nanoparticle synthesis, plant leaf extract has been used to synthesize magnesium nanoparticles using a biological route. FeCl_3_ solution was prepared by dissolving ferric chloride hexahydrate crystals (Thomas Baker’s). It was used to etch the copper plate and deposit the circuit on the board. The probes were made and cut into small pieces using the cutter. The probes were used in chemical vapor sensing. All the glassware used was of Borosil glass (Mumbai, India). The glacial acetic acid, ammonia, and methanol were used in small quantities (20 mL) for chemical vapor sensing.

### 2.2. Methodology

**Figure 1:**
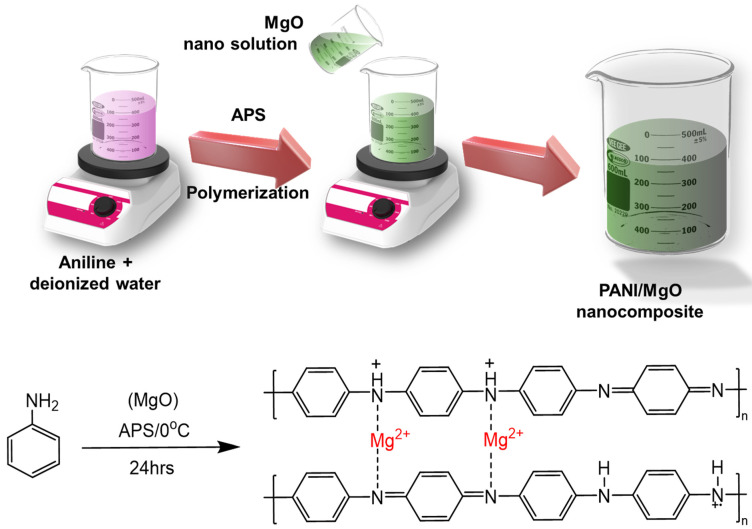
Synthesis of the PANI–magnesium oxide composite. The formation of Mg(II)-doped PANI is illustrated, with Mg acting as the interlink between two polymeric chains during polymerization. However, in a broader context, the resulting structure is referred to as a MgO/PANI composite, Reproduced with permission from ref. [21], Patil, V.B et al., Polymers. Adv. Technol, Wiley Publisher, 2021.

The preparation is divided into three categories: likely preparation of polyaniline, preparation of magnesium nanoparticle solution, and the preparation of the PANI/MgO polymer nanocomposite material. The preparation is discussed in Figure 1. The aniline solution of one molarity was taken in a volumetric flask and mixed with distilled water. Hydrochloric acid was added to the aniline solution in the same weight ratio to dissolve the aniline into the distilled water [6,7]. APS was prepared as a 1 M solution by dissolving it into distilled water. The aniline solution was kept on a magnetic stirrer with continuous stirring at 400 rpm, and then the APS solution was added drop-by-drop to the aniline solution. After completely adding the APS solution to the aniline solution, the solution was stirred for one hour to complete the polymerization.

### 2.3. Preparation of the Magnesium Oxide Nanoparticles

Magnesium nanoparticles were synthesized using a green approach, leveraging biological materials for nanoparticle formation. This method uses magnesium sulfate (MgSO_4_) as the metal precursor. A magnesium sulfate solution was prepared by dissolving the salt in distilled water to achieve a concentration of 1 M. The biological reducing agent was derived from plant leaves. Fresh leaves were collected, washed thoroughly to remove impurities, and cut into small pieces. These leaf pieces were subjected to extraction by boiling them in distilled water, resulting in a plant extract rich in phytochemicals that can act as reducing and stabilizing agents. Once the plant extract was prepared, it was gradually added to the magnesium sulfate solution under constant stirring to ensure thorough mixing. The reaction mixture was then exposed to a specific radiation source to enhance the reduction process. During this process, the color of the solution changed, which is a visual indication of the formation of the magnesium nanoparticles. The observed color change is attributed to the surface plasmon resonance effect, which occurs when nanoparticles form and exhibit unique optical properties. This nanoparticle synthesis method is advantageous due to its eco-friendliness, cost-effectiveness, and potential for large-scale production. Using plant extracts as reducing agents eliminates the need for harmful chemicals, aligning with green chemistry principles. The resultant magnesium nanoparticles could have significant applications in various fields, including medicine, electronics, and catalysis [11,12].

### 2.4. Preparation of the Polyaniline/MgO Polymer Nanocomposite

The polyaniline (PANI) solution was stirred continuously for one hour to ensure homogeneity. A measured amount of magnesium nanoparticles was then gradually added drop-by-drop to the stirred solution, ensuring proper mixing and uniform distribution of the nanoparticles. The nanoparticles were added at various weight percentages, specifically 0.1%, 0.2%, and 0.3%, to create different composite ratios. After the complete addition of the nanoparticles, the resulting mixture was filtered to separate the composite material. The solid deposits collected on the filter, comprising the PANI/MgO composite material (Figure 1), were then dried. The dried composite material was ground into a fine powder to ensure uniformity and prepare it for subsequent analysis. The fine powder of the PANI/MgO composite was subjected to various characterization techniques to evaluate its properties and potential applications.

## 3. Characterizations

The precipitated PANI/MgO composite material was characterized using advanced analytical techniques. Scanning Electron Microscopy (SEM) coupled with Energy-Dispersive X-ray Analysis (EDAX) was performed using the JEOL JSM-6360 model. This high-resolution SEM operated at 30 kV with a resolution of 3.0 nm, allowing for detailed imaging of the composite’s surface morphology and elemental composition analysis. X-ray Diffraction (XRD) analysis was carried out using a Philips-3710 polycrystalline diffractometer. The XRD measurements were performed over a 2θ range of 10° to 100° using CuKα1 radiation (λ = 1.54056 Å) to identify the crystalline phases present in the composite and to determine the crystallographic structure. Fourier Transform Infrared Spectroscopy (FTIR) investigated the composite functional groups of PANI and molecular interactions. The FTIR analysis was performed over a spectral range from 4000 cm⁻^1^ to 450 cm⁻^1^ using a Perkin Elmer Spectrum One spectrophotometer. This technique provided insights into the chemical bonding and the presence of specific functional groups within the composite. These characterizations were carried out at Shivaji University, Kolhapur. The findings from these analyses are presented in the following sections of this report [15,19].

## 4. Result and Discussions

### 4.1. FTIR

**Figure 2:**
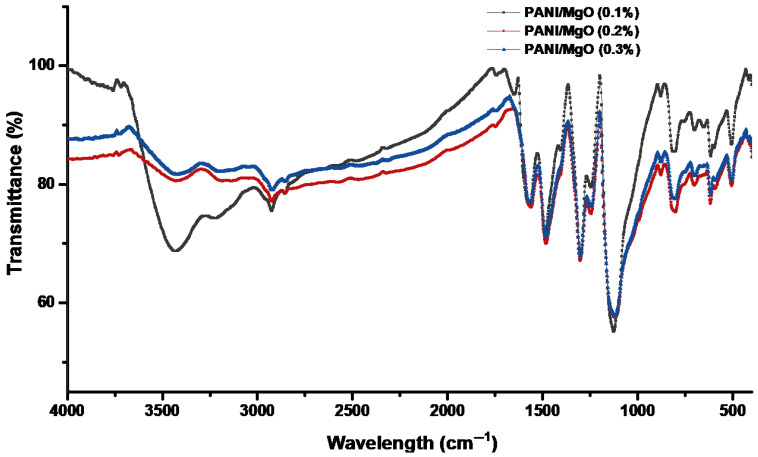
FTIR curve of sample PANI/MgO nanocomposites.

The FTIR spectra of the PANI/MgO composite samples with various concentrations of magnesium nanoparticles (0.1%, 0.2%, and 0.3%) are shown in Figure 2, where the observed peaks correspond to specific functional groups and interactions within the material. The spectral line at 3428.88 cm⁻^1^ indicates an interaction between the magnesium nanoparticles and the polyaniline (PANI) matrix, which is attributed to hydrogen bonding between the magnesium and the N-H groups of PANI and becomes more pronounced at the 0.3% concentration of magnesium nanoparticles. The peak at 506 cm⁻^1^ corresponds to C-N-C bonding, signifying the stability of covalent bonding within the benzene ring structure of the PANI. The wavenumber at 2924.98 cm⁻^1^ is associated with the =CH_2_ group, indicating an aliphatic character within the polymer chain. In comparison, the band at 1570.12 cm⁻^1^ illustrates the elongation of the quinoid ring, represented by the –C=N– stretching vibration, characteristic of the conjugated structure within the polymer. The peak at 1488.36 cm⁻^1^ corresponds to the benzenoid ring’s C–N stretching vibrations, highlighting the synthesized aromatic nature of PANI. The band at 1127.32 cm⁻^1^ indicates S=O stretching, suggesting the presence of camphor–sulfonic acid groups bonded to the polymer molecule, which may enhance the polymer’s conductivity. Finally, the wavenumber at 803 cm⁻^1^ designates out-of-plane C-H bending vibrations within the benzenoid ring, further confirming the aromatic nature of the polymer. These FTIR results provide crucial insights into the molecular interactions and structural integrity of the PANI/MgO composite as the concentration of magnesium nanoparticles varies [21,22].

### 4.2. SEM Analysis

**Figure 3:**
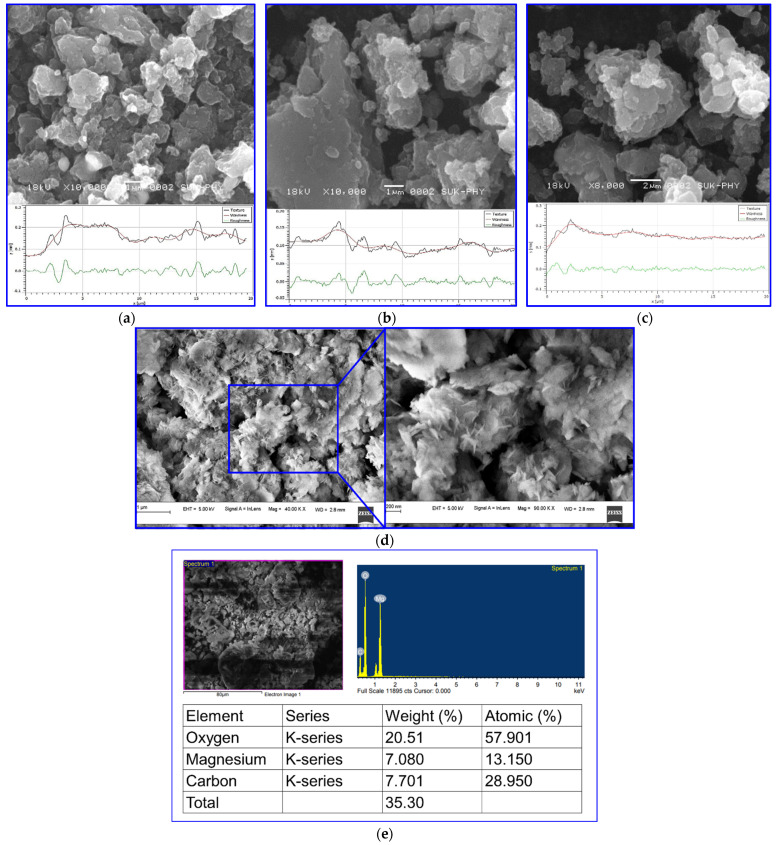
(**a**) SEM image and surface studies of PANI/MgO (0.1%), (**b**) PANI/MgO (0.2%), (**c**) PANI/MgO (0.3%) composites; (**d**) pure MgO nanoparticles; and (**e**) EDS of the MgO nanoparticles.

The surface characteristics of the PANI/MgO nanocomposite, including texture, waviness, and roughness, were meticulously analyzed using a profilometer. These surface parameters are critical in defining the distribution and morphology of particles across the material’s surface. As depicted in Figure 3, the profilometric analysis reveals a maximum peak height of 2.6 μm, indicating significant surface irregularities. In this context, surface roughness provides valuable insights into the unevenness of the material over a specified range, with the analysis focusing on the 0–15 μm scale. The data indicate a considerable surface unevenness, characterized by numerous gaps and asperities. Further examination of the PANI/MgO nanocomposite surface morphology shows an irregular structural pattern, with the polymer matrix consisting of both large and small grains distributed sparsely across the surface. The smallest observed grain size was approximately 40 nm, while these grains aggregated to form larger structures, with the largest grain size reaching up to 200 nm, as illustrated in Figure 3. Upon doping the composite in the neutral state of the conducting polymer (CP), the particle size of the polymer increases, leading to the formation of larger molecular assemblies on the order of 5 μm. However, the dopant was not uniformly distributed, resulting in observable gaps between adjacent particles, with these gaps measuring approximately 3 μm. As the concentration of magnesium nanoparticles in the composite increased, a corresponding rise in the aggregation of dopant particles around the parent polymer molecules was observed. This increase in dopant concentration led to a more uniform surface thickness due to the accumulation of dopants, which influenced the overall surface characteristics. Specifically, the waviness and roughness of the surface tended to decrease as the concentration of magnesium nanoparticles increased. Additionally, a marked increase in particle size was noted with varying concentrations of the nano-Mg dopant, further influencing the surface morphology and material properties [35].

### 4.3. XRD Studies

**Figure 4:**
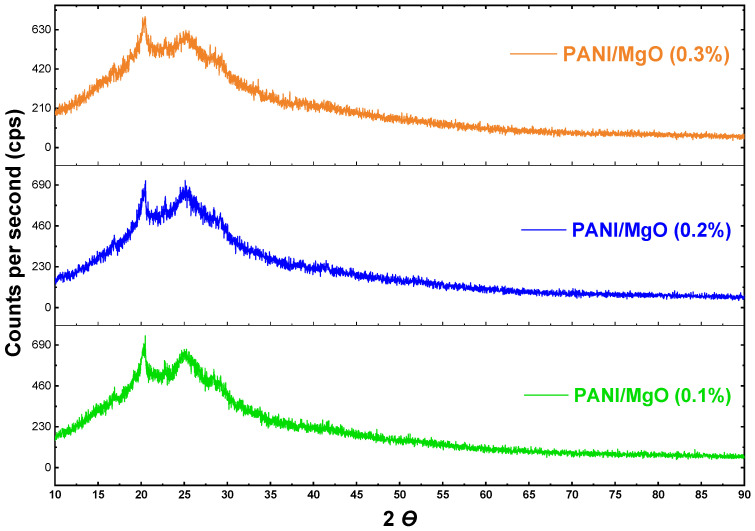
XRD curves for the PANI/MgO nanocomposites.

The XRD spectra for the PANI/MgO samples with various concentrations of Mg nanoparticles are presented in Figure 4. The diffraction peaks observed at 2θ values of approximately 20° and 25° are attributed to the π-conjugation in the polyaniline (PANI) matrix, indicating the presence of semi-crystalline regions within the sample. These peaks also suggest a lack of aggregation in the polymer chains, particularly in the benzenoid and quinonoid groups of the PANI backbone [20]. For the sample with 0.1% Mg nanoparticle addition, the interplanar spacing (*d_hkl_*) calculated from the peak at 2θ = 20.5° and 24.84° is 7.49 Å, with an atomic spacing of 4.33 Å along the (111) planes. When the Mg nanoparticle concentration is increased to 0.2%, the *d_hkl_* values shift slightly to 7.57 Å, with an atomic spacing of 4.39 Å, corresponding to 2θ peaks at 20.1° and 24.96°. Further increasing the dopant concentration to 0.3% results in a *d_hkl_* of 7.64 Å and an atomic spacing of 4.41 Å, with corresponding 2θ peaks at 20.2° and 25.12°. The XRD analysis demonstrates that with increasing Mg nanoparticle concentration, there is a corresponding increase in the interplanar spacing and the overall atomic size of the PANI/MgO composite. This trend, corroborated by the observed distribution of particles in the SEM images, confirms that the incorporation of magnesium nanoparticles leads to an increase in the semi crystalline structure’s atomic dimensions within the polyaniline matrix [36].

## 5. Application Studies

### 5.1. Chemical Vapor Sensor

The synthesized PANI/MgO polymer nanocomposite material has been specifically developed for application in chemical vapor sensing. A series of probes were meticulously prepared to evaluate its performance in this application. The preparation process involved uniformly coating the surface of the probes with the PANI/MgO nanocomposite material, ensuring consistent material coverage and optimal interaction with the target chemical vapors. Once the probes were coated, their electrical resistance was measured under controlled exposure to specific chemical vapors. This step was critical in assessing the material’s sensitivity and responsiveness to vapor presence. The change in resistance, induced by the interaction between the chemical vapors and the PANI/MgO composite, was monitored in real-time. This allowed for a detailed analysis of the sensing capabilities of PANI, including its selectivity, response time, and overall effectiveness in detecting the presence of various chemical vapors. The ability of the PANI/MgO nanocomposite to undergo a measurable change in resistance upon exposure to chemical vapors underscores its potential as a highly effective sensing material. These results validate the utility of the PANI/MgO composite in practical sensing applications and provide critical insights into its operational mechanisms, laying the groundwork for further development and optimization of advanced chemical vapor sensors.

### 5.2. Preparation of Sensor Probes

**Figure 5:**
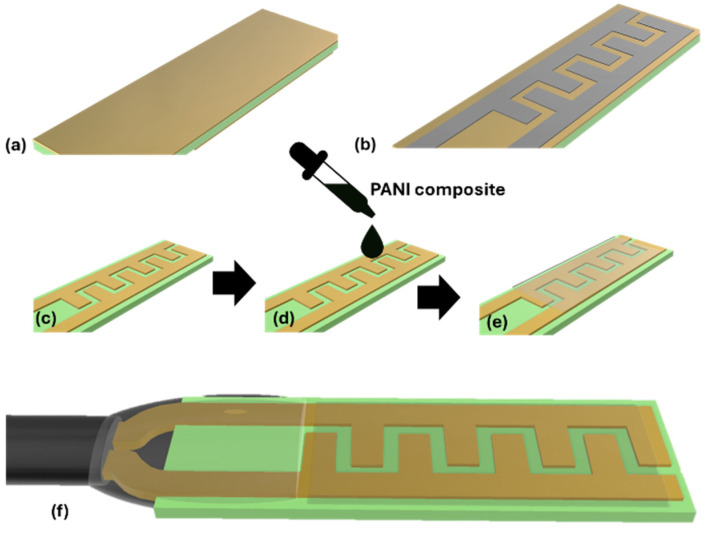
Sensor probe preparation: (**a**) Copper clad board (CCB), (**b**) sensor probe pattern printed on CCB, (**c**) sensor probe pattern etched using ferric chloride (FeCl₃), (**d**) sensor probe coated with PANI nanocomposite, (**e**) sensor dried at room temperature, (**f**) final prepared sensor probe.

The probes used were prepared by using copper cladded circuit boards. The required design of the circuit is marked on the board. Then, the board is dipped into the ferrous chloride solution to etch the unwanted part of the board, as shown in Figure 5. To complete the etching process, the board is kept in the solution for 24 hr (see Figure 5). After the etching process, the probes were cut into small strips. Then, the working probes were coated with the powder form of the PANI/MgO material. As shown in Figure 5c, after coating the probes with the PANI/MgO material, they are placed in the prepared vacuum chamber where the chemical vapors are passed through, and the probe’s resistance is checked over time. The following figures show the vacuum chamber setup and mounting of the probes into the setup. The resistance vs. time graphs were drawn and analyzed to check the variation in the resistance [37].

### 5.3. Sensing Results

**Figure 6:**
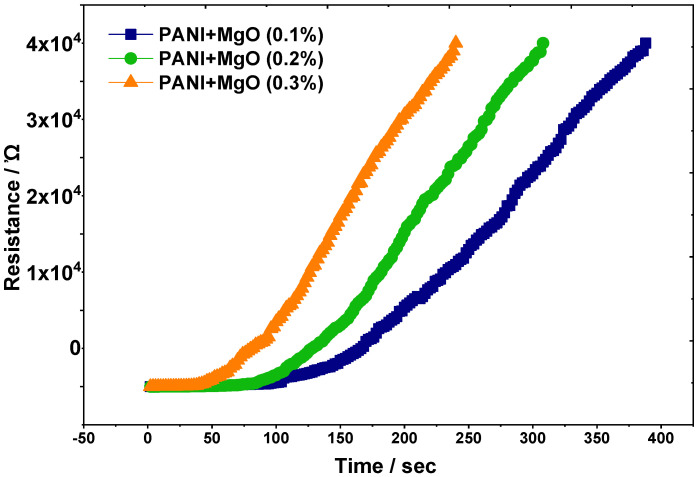
Results of sensing of ammonia vapors employing PANI/MgO composite with different concentrations of MgO (in %).

The resistive behavior of the PANI/MgO polymer nanocomposite material was systematically evaluated in the presence of ammonia vapor. As illustrated in Figure 6, the resistance of the testing strips increased progressively with exposure time, indicating an active interaction between the composite material and the ammonia vapor. Notably, strips containing a higher concentration of the MgO nanoparticles exhibited a more pronounced increase in resistance, suggesting that these strips function as superior absorbers and exhibit stronger interactions with the chemical vapor. This observation highlights the enhanced sensitivity of the PANI/MgO nanocomposite, which correlates positively with the increased loading of magnesium oxide nanoparticles. The higher MgO concentration within the composite facilitates more effective adsorption of the ammonia molecules, thereby contributing to a greater change in resistance. Furthermore, the testing strips demonstrated excellent reusability, as repeated sensing cycles yielded consistent results, underscoring the reproducibility and stability of the nanocomposite in chemical vapor sensing applications. These findings suggest that the PANI/MgO nanocomposite, with its tunable sensitivity and robust performance, holds significant potential for use in ammonia detection and possibly other chemical vapor sensing technologies. The reproducibility of the strips further supports their practical applicability in real-world sensing environments, offering a reliable and reusable solution for monitoring chemical vapors.

#### Ammonia Vapor Sensing

**Figure 7:**
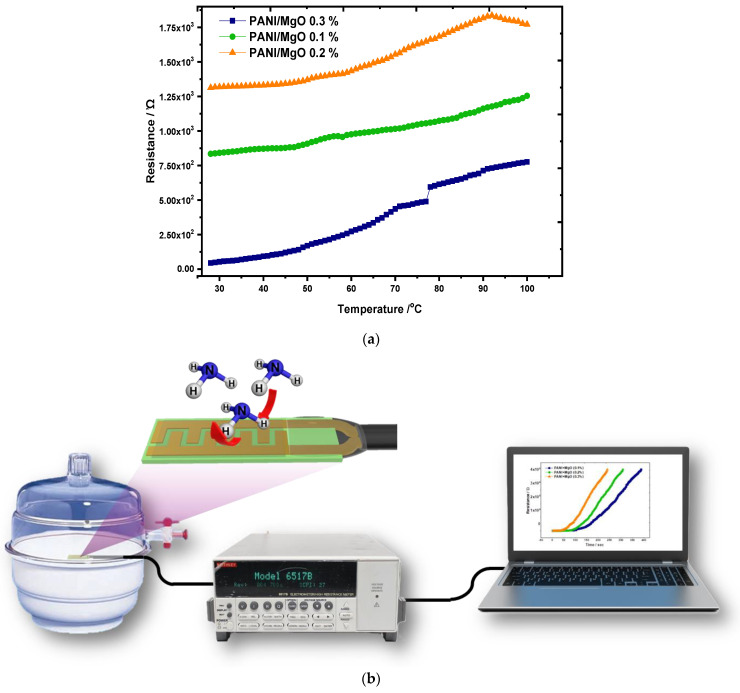
(**a**) DC conductivity of PANI/MgO Composites (**b**) sensor setup.

Polyaniline (PANI) emeraldine base, as depicted in Figure 7, serves as the fundamental chemical building block for the sensor. When incorporated into the PANI/MgO composite, which acts as a strong Lewis acid, the material reacts with ammonia gas to produce ammonium ions. These ions are derived from the delocalized H⁺ at the PANI end, forming the pernigraniline base compound. The resultant pernigraniline base is an aniline-rich, unstable PANI derivative. Due to its hydrogen adsorption capabilities, this compound can be recycled back into the emeraldine base, making it reusable [40]. The sensor’s response shows increased data output, which can be attributed to the chemical equilibrium between the emeraldine salt and the pernigraniline base at a constant concentration of ammonia vapors. Ammonia, being a strong base, rapidly oxidizes to form a complex when interacting with a strongly acid-doped material like PANI/MgO. This interaction also indicates that the rate of gaseous ammonia molecules’ interaction with conducting polymers (CPs) was measured to assess the effectiveness of chemoresistance. The observed reduction in resistance with increasing ammonia concentration demonstrates that conductivity increases accordingly. Additionally, the activation time for electrical conductivity decreases from 100 s to 47 s, as can be estimated from the graph.

### 5.4. DC Conductivity

Figure 7 clearly illustrates that the PANI/MgO composite exhibits a positive temperature coefficient of resistance up to a critical temperature, after which it transitions to a negative coefficient of resistance beyond 85 °C. The chemical bonds formed in the PANI doped with Mg involve weak electrostatic van der Waals interactions and ionic bonds. These bonds are relatively weak and require minimal thermal activation energy to rupture. Consequently, the valence electron inter-ionic repulsions lead to the formation of subatomic particles such as polarons and bipolarons, which affect the charge carrier population and decrease conductivity once the peak temperature is reached. Since magnesium typically exists in an oxidized state of +2, it can donate two electrons to the conduction band as the temperature increases. At lower concentrations of Mg, such as 0.1%, the conductivity remains relatively low. However, as the dopant concentration increases in increments of 0.1%, an enhancement in conductivity is observed. Specifically, at a 0.2% Mg dopant concentration, the conductivity significantly increases between 80 °C and 90 °C. Further increasing the Mg concentration to 0.3% results in a notable rise in conductivity, with the critical temperature point shifting to approximately 76 °C.

### 5.5. Comparative Studies of Sensors

Table 1 compares key sensing parameters for the latest ammonia gas detection technologies. It highlights how materials like graphene-based transistors, polyaniline/MoS_2_/phosphorus-doped graphene nanocomposites, and plasmonic nanostructures have advanced the field. These materials show major improvements, particularly regarding detection limits, response times, and selectivity. Some sensors now detect ammonia at levels as low as 0.01 ppm. Response times range from nearly instant to about 60 s, with most sensors working efficiently at room temperature. Selectivity remains crucial, especially in complex environments, and nanocomposites tend to offer superior performance. These advancements are key to improving ammonia detection for industrial and environmental applications.

## 6. Conclusions

In the present study, PANI/MgO nanocomposites were successfully synthesized using an in-situ oxidative polymerization method with varying concentrations of magnesium nanoparticles. These magnesium nanoparticles were produced via a green synthesis process, and their formation was confirmed through FT-IR analysis and SEM imaging. SEM analysis further revealed the uniform distribution of Mg nanoparticles within the composite matrix. The PANI/MgO nanocomposites significantly enhanced both sensing and electrical conductivity properties compared to pure PANI. Unlike the PANI sensor, the PANI/MgO-based chemosensor rapidly responded to ammonia vapors. It was also shown that the Mg concentration within the sensor directly affects its response time, sensitivity, and reproducibility, with the results indicating optimal performance at specific concentrations. However, it was observed that the polymer nano-composite could not be useable few trials, not continuous in sensing applications due to its inherent properties. Despite this limitation, the polymer composite material has substantially impacted the sensor industry and holds the potential for recycling in future research toward electronic sensing noses. The findings of this study contribute to the development of advanced electronic noses for chemical vapor detection, paving the way for future innovations in sensor technology.

## Figures and Tables

**Table 1:** Performances of different sensors.

Sensing Material	Detection Limit in ppm	Response Time in Seconds	Recovery Time in Seconds	Operating Temperature in °C	Detection Range in ppm	Ref.
Catalytic, metal oxide, polymer-based	1–50 ppm	10–60	40–120	25–300 °C	1–100	[45]
Graphene/semiconductor and FET sensors	~1 ppm	5–15	10–20	~25 °C	0.1–100	[46]
PANI/MoS2/PGO nanocomposite	0.01 ppm	12	30	~25 °C	10–100	[47]
Plasmonic nanostructures	8.5 ppm	Instant (color change)	N/A	~25 °C	8.5–100	[48]
Various gas sensor arrays	0.1–50 ppm	5–30	10–60 s	~25 °C	0.1–100	[29,48]
PANI/MgO nanocomposite	30 ppm	10	16	~27 °C	30–100	Present work

## Data Availability

The original contributions presented in the study are included in the article, further inquiries can be directed to the corresponding author.
